# Separation of Spacecraft Noise From Geomagnetic Field Observations Through Density‐Based Cluster Analysis and Compressive Sensing

**DOI:** 10.1029/2022JA030757

**Published:** 2022-09-15

**Authors:** Alex Paul Hoffmann, Mark B. Moldwin

**Affiliations:** ^1^ Climate and Space Sciences and Engineering University of Michigan Ann Arbor MI USA

**Keywords:** magnetometer, noise cancellation, cocktail party problem, CubeSat, spacecraft, instrumentation

## Abstract

The use of magnetometers for space exploration is inhibited by magnetic noise generated by spacecraft electrical systems. Mechanical booms are traditionally used to extend magnetometers away from noise sources. If a spacecraft is equipped with multiple magnetometers, signal processing algorithms can be used to compare magnetometer measurements and remove stray magnetic noise signals. We propose the use of density‐based cluster analysis to identify spacecraft noise signals and compressive sensing to separate spacecraft noise from geomagnetic field data. This method assumes no prior knowledge of the number, location, or amplitude of noise signals, but assumes that they have minimal overlapping spectral properties. We demonstrate the validity of this algorithm by separating high latitude magnetic perturbations recorded by the low‐Earth orbiting satellite, SWARM, from noise signals in simulation and in a laboratory experiment using a mock CubeSat apparatus. In the case of more noise sources than magnetometers, this problem is an instance of underdetermined blind source separation (UBSS). This work presents a UBSS signal processing algorithm to remove spacecraft noise and minimize the need for a mechanical boom.

## Key Points

- We present the first use of compressive sensing with cluster analysis to separate spacecraft noise from geomagnetic field data
- We demonstrate the separation of wideband noise signals in simulation and in a lab experiment using SWARM residual geomagnetic field data
- The method enables accurate magnetic field measurements from resource limited and magnetically noisy spacecraft such as boomless CubeSats

## 1 Introduction

Spacecraft equipped with magnetometers can be used to capture in situ measurements of magnetic phenomena in the geospace environment. These measurements are necessary to answer key questions about the nature of the Earth's magnetosphere and its interaction with interplanetary magnetic fields. Understanding how the heliosphere directs the flow of energy, mass, and momentum between the Sun and Earth is critical for applications such as space weather modeling, space exploration, and climate science. A number of missions use spacecraft equipped with magnetometers to measure magnetic fields. For example, The European Space Agency's SWARM mission uses a constellation of three satellites to provide high fidelity magnetic field measurements used to model the Earth's magnetic field and study the Earth's dynamo (Fratter et al., 2016). Magnetometers provide invaluable data for space science research, however, the quality of the data are often limited by magnetic noise generated by the spacecraft. Electrical systems onboard a spacecraft generate stray magnetic fields that interfere with magnetic field measurements. The strength of magnetic fields in the geospace environment ranges several orders of magnitude with natural phenomena such as the interplanetary magnetic field occurring on the order of 6 nT to the Earth's magnetosphere in low‐Earth orbit measuring on the order of 60,000 nT. Spacecraft subsystem magnetic fields may completely eclipse the perturbations in natural magnetic fields which are of interest to understanding waves and currents in the solar wind and magnetosphere. The presence of these stray magnetic fields is a significant obstacle for missions that utilize magnetic field data (Ludlam et al., 2009; Russell, 2004).

On satellites, stray magnetic fields can be generated by subsystems such as solar panels, reaction wheels, battery currents, and magnetorquers. The magnetometer on the CubeSat, Ex‐Alta 1, recorded magnetic field noise generated by a magnetorquer which exceeded 7,500 nT peak‐to‐peak (Miles et al., 2016). Satellite magnetometers are typically fixed at the end of a mechanical boom to reduce the magnitude of noise generated by the spacecraft. For example, the mission SWARM uses two magnetometers mounted on a 4.3 m boom (McMahon et al., 2013). However, the use of a boom is not always possible in designs such as rovers and CubeSats where gravity and cost are limiting factors. Additionally, the implementation of a boom does not always guarantee the elimination of spacecraft noise from magnetic field measurements. The spacecraft, DMSP, employs a single magnetometer on the end of a 5 m boom, but still faces issues with spacecraft noise (Kilcommons et al., 2017).

The use of a single magnetometer on a spacecraft requires a careful magnetic cleanliness design and characterization of the spacecraft's magnetic signature in order to minimize stray magnetic fields. In the case of the spacecraft Cassiope, a software update changed the behavior of the spacecraft's fluxgate magnetometer (MGF). Special spacecraft maneuvers to decrease the spacecraft's noise signature were required in order to recalibrate the MGF (Miles et al., 2019). Algorithms to autonomously identify spacecraft noise would allow Cassiopie to do in situ MGF calibration without special spacecraft maneuvers.

In spacecraft with multiple magnetometers, the traditional way to cancel stray magnetic field noise is to perform gradiometry. Gradiometry is a technique which compares magnetometer signals from two spatially separated sensors and calculates the gradient of between them. Ness et al. (1971) uses the gradient to fit a dipole to the spacecraft noise and formulate a coupling matrix. The coupling matrix is then used to subtract the spacecraft noise from the magnetometer measurements. This method can also be applied to higher order magnetic fields but requires arduous pre‐flight characterization of the spacecraft's magnetic signature. Ream et al. (2021) uses gradients in the frequency domain to identify and suppress spacecraft noise. However, this method assumes that the spectra of the ambient magnetic field and the spacecraft noise do not overlap. Pope et al. (2011) uses the axial gradients and fuzzy logic to identify spacecraft noise according to the subsystem that generates it. The identified noise segments are then corrected in the time domain using information about the noise generated by the subsystem. This method is successful at identifying and removing many different individual noise sources, however, it is not designed to correct multiple concurrent noise sources.

Other noise cancellation methods employ state estimation of the magnetic fields generated by spacecraft subsystems by examining spacecraft housekeeping data. Deshmukh et al. (2020) uses a supervised machine learning algorithm in order to estimate the transfer function of housekeeping currents to stray magnetic fields. Total knowledge of a spacecraft's magnetic signature would allow for perfect interference cancellation, however, housekeeping telemetry provides an incomplete mapping of a spacecraft's current distribution. Additionally, housekeeping data are often sampled at a low cadence and may not have the appropriate bandwidth to identify higher frequency noise. For low cost applications with a large number of spacecraft, such as CubeSat constellations, it is advantageous to use an algorithm that does not require a boom, rely on prior knowledge of the spacecraft's magnetic signature, or requires human analysis.

Recent progress has been made in magnetometer noise cancelation through the application of blind source separation (BSS) algorithms. BSS is the separation of a mixture of source signals without prior knowledge of the signal type or magnetometer location. Constantinescu et al. (2020) use maximum variance analysis (MVA) to clean spacecraft magnetometer data. The MVA algorithm finds an orthogonal set of axes to maximize the variance of the measured signals. These axes represent the principle components which are used to identify and remove noise sources. This application of MVA requires that the variance in the noise sources is larger than the variance in the background magnetic field, and can only identify a limited number of noise signals. Imajo et al. (2021) proposed the use of independent component analysis (ICA) to separate geomagnetic field data, captured by the satellite Michibiki‐1, from stray magnetic field noise. This algorithm separates signals based on statistical independence, and works well when the number of noise sources are not more than the number of magnetometers (Naik & Kumar, 2009). The MVA and ICA algorithms both separate signals through optimizing statistical quantities, however, they are limited by the number of noise signals they can identify. Sheinker and Moldwin (2016) proposed a novel BSS algorithm that uses an analytical formulation to estimate the gain of a single noise source between magnetometers. This method is designed for the case in which a single noise source is present, and does not account for the presence of multiple noise sources. Although, the method may be adapted to remove multiple noise sources by adding more magnetometers.

In this work, we present the application of an underdetermined blind source separation (UBSS) algorithm based on the unsupervised machine learning algorithm, Density Based Spatial Clustering of Applications with Noise (DBSCAN), and compressive sensing to separate the ambient magnetic field from spacecraft noise. UBSS is a class of problems in which there are *M* sensors and *N* unknown source signals such that *M* < *N*. The *M* sensors, defined by the complex signals $B\left(k\right)\in C^{M}$, contain a mixture of the N source signals, defined by $S\left(k\right)\in C^{N}$. At the frequency bin, *k*, the source signals combine in an unknown mixing matrix $K\in C^{M\times N}$. In UBSS, no prior knowledge of the source signals is assumed and the number of source signals that can be separated is not limited by the number of sensors. The system used to model UBSS is defined by the following relationship.

(1) B(k)=KS(k)

UBSS is a topic that has been thoroughly researched in other fields such as acoustics and radar signal processing. In the field of acoustics, this problem is famously referred to as the cocktail party problem. In the cocktail party problem, there is a room full of people each having conversations. An array of microphones is placed in the room to record the concurrent conversations. The microphone recordings are then used to separate each individual voice. Guo et al. (2017) demonstrate the identification of four human voices using three microphones. He et al. (2021) also demonstrate the separation of six flutes recorded by three microphones using the DBSCAN algorithm.

Due to the spatial structure of magnetic fields, the same algorithms developed to solve the cocktail party problem cannot be directly applied to magnetic noise cancelation. When considering a dipole noise source, the vector magnetic field will have a different magnitude and polarity depending on the magnetic latitude and radial distance of the magnetometer. In this work, we model the spatial structure of magnetic fields with a phase, although magnetic noise signals mix instantaneously. The structure of the magnetic noise signal is not always dipolar, and will change depending on the geometry of the noise source. In magnetic UBSS, the mixing matrix, *K*, is a complex matrix representing the gain and phase of each signal at each magnetometer. In radar signal processing, Bai et al. (2021) apply a similar approach by using complex mixing matrices to model time‐delayed radar signals with different directions of arrival. In this work, we use DBSCAN to estimate the mixing matrix, *K*. Once *K* is known, compressive sensing is used to restore the geomagnetic field signal from the noisy magnetometer data.

We present two experiments to validate this algorithm. The first experiment separates four computer‐simulated noise signals from an ambient magnetic field signal. The second experiment separates the same ambient magnetic field signal using real magnetic field data recorded using an experimental CubeSat apparatus with copper coil‐generated signals and three PNI RM3100 magnetometers (Regoli et al., 2018). The aim of this work is to develop a robust signal processing algorithm to remove spacecraft noise and minimize the need for a mechanical boom or a magnetically clean spacecraft. This work focuses on developing a noise cancellation algorithm for geomagnetic field data, but can also be applied to remove noise in measurements of planetary magnetospheres and interplanetary magnetic fields.

## 2 Methodology

We apply a two step approach to removing spacecraft noise and reconstructing the ambient magnetic field. The first step is to discover the mixing matrix, *K*, defined in Equation 1. This is achieved by preprocessing the magnetometer data into a clusterable form and applying a clustering algorithm. The second step is to reconstruct the ambient magnetic field and noise signals using compressive sensing. In this step, the mixing matrix, *K*, is used to demix the magnetometer signals via convex optimization. This two‐step process is designed to be applied to each magnetometer axis separately.

### 2.1 Signal Preprocessing

The separation of magnetic field signals from stray magnetic fields is analogous to a problem thoroughly researched in other fields such as acoustics and is called UBSS. This problem has been heavily investigated for microphone and radar arrays, but the unique spatial structure of magnetic fields introduces new complications which have not been well‐researched. When considering a dipole noise source, the placement of magnetometers at different magnetic latitudes alters the magnitude and polarity of the noise signal. We model this effect as a phase, despite the noise sources mixing instantaneously. The time‐frequency (TF) domain mixing model, *B*(*t*,*k*) = KS(*t*,*k*), is defined by the following system:

(2) $$\left[\begin{array}{c} B_{1}\left(t,k\right)\\ B_{2}\left(t,k\right)\\ \vdots \\ B_{m}\left(t,k\right) \end{array}\right]=\left[\begin{array}{ccccc} 1 & k_{12}∠\phi_{12} & k_{13}∠\phi_{13} & \ldots & k_{1n}∠\phi_{1n}\\ 1 & k_{22}∠\phi_{22} & k_{23}∠\phi_{23} & \ldots & k_{2n}∠\phi_{2n}\\ \vdots & \vdots & \vdots & \ddots & \vdots \\ 1 & k_{m2}∠\phi_{m2} & k_{m3}∠\phi_{m3} & \ldots & k_{mn}∠\phi_{mn} \end{array}\right]\left[\begin{array}{c} S_{1}\left(t,k\right)\\ S_{2}\left(t,k\right)\\ \vdots \\ S_{n}\left(t,k\right) \end{array}\right]$$

In this mixing system, the signals *S*_*i*_(*t*, *k*) are the source signals at time *t* and frequency *k*. The ambient magnetic field signal we seek to recover, *S*_1_(*t*, *k*), is assumed to be identical at each magnetometer and is represented by a column of ones in the mixing matrix. In the geospace environment, this allows us to observe phenomena such as ultralow frequency (ULF) waves which have frequencies less than 5 Hz (Jacobs et al., 1964). The phases, *ϕ*_*ij*_ = {0, *π*}, in the mixing matrix, *K*, account for the difference of a signal seen by magnetometers at different magnetic latitudes. The phase, *ϕ*_*ij*_, is determined by the spatial structure of the noise signal, which depends on the geometry of the noise source. This model does not require that noise sources be dipolar. The value in the mixing matrix *k*_*ij*_ *∠ϕ*_*ij*_ represents the complex value $k_{ij}e^{j\phi_{ij}}$. This value defines presence of the signal *S*_*j*_(*t*, *k*) at magnetometer *B*_*i*_(*t*, *k*).

Sparsity is a precondition of both mixing matrix estimation and compressive sensing, however, spacecraft noise signals are not often sparse in the time domain. The magnetometer signals, *b*(*t*), are transformed into the TF domain using a Fourier transform in order to increase signal sparsity. Typically, the Short‐Time Fourier Transform (STFT) is used because signals that are present in multiple time windows will provide more data points to be clustered [id = Revision Two]. As a result, periodic signals are easier to identify and remove than aperiodic signals. However, aperiodic signals can be separated with sufficient TF resolution. In this work, we use the Non‐Stationary Gabor Transform (NSGT) to transform magnetometer signals into the TF domain. NSGT has advantages over the STFT because it allows the user to evolve the window size with respect to frequency (Holighaus et al., 2013). As a result, high and low frequencies are not limited to the same window size, and frequency resolution is constant across the frequency spectrum. In order to apply the NSGT, the user specifies a quality, *Q*, and the lowest frequency they would like to observe. The parameter, *Q*, is used to automatically calculate the window size with respect to the desired frequency resolution. In comparison to the STFT, the NSGT provides finer frequency resolution at low frequencies and better time resolution at higher frequencies. We perform the NSGT to obtain the UBSS model *B*(*t*, *k*) = KS(*t*, *k*). The mixing system of a sparse TF bin where only the signal, S_*j*_(*t*,*k*), is present can be defined by a single mixing vector:

(3) $$\left[\begin{array}{c} \left|B_{1}\left(t,k\right)\right|\\ \left|B_{2}\left(t,k\right)\right|\\ \vdots \\ \left|B_{m}\left(t,k\right)\right| \end{array}\right]=\left[\begin{array}{c} k_{1j}\\ k_{2j}\\ \vdots \\ k_{mj} \end{array}\right]|S_{j}\left(t,k\right)|$$

The operator |*a* + *jb*| applied to the complex value *a* + *jb* returns the magnitude of complex value, $\sqrt{a^{2}+b^{2}}$. Equation 3 can be rewritten element‐wise as:

(4) $$|S_{j}\left(t,k\right)|=\frac{\left|B_{1}\left(t,k\right)\right|}{k_{1j}}=\frac{\left|B_{2}\left(t,k\right)\right|}{k_{2j}}=\ldots =\frac{\left|B_{m}\left(t,k\right)\right|}{k_{mj}}$$

**Figure 1:**
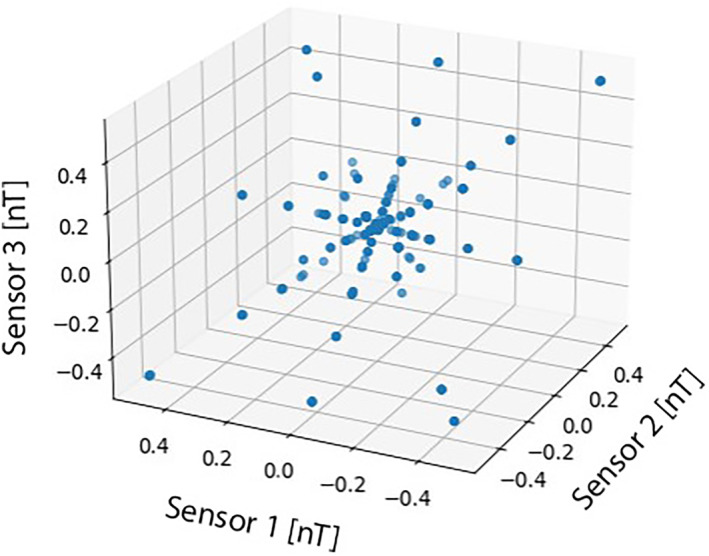
Three magnetometer measurements of six computer‐simulated sinusoidal noise signals. Each magnetometer signal is transformed into the time‐frequency (TF) domain using the STFT. The magnitude of the three resulting TF signals are taken and plotted against each other in a scatter plot. The scattered TF points from each magnetometer form straight lines due to Equation 4. This figure does not include the phase subdomain of the **H**‐domain.

Equation 4 is equivalent to the symmetric form of a line with slope defined by the mixing vector of the noise signal. In order to find the mixing vector of a noise signal, we define a TF space $H\in R^{2m}$ in which each phase and magnitude of the *m* magnetometer signals are an axis. Sparse TF points will draw straight lines through the origin in the **H**‐domain with a slope proportional to the signal's mixing vector. Figure 1 shows an example of a scatter plot of three mixed TF signals composed of six source signals. The mixed signals form straight lines with slopes defined by Equation 4.

### 2.2 Mixing Matrix Estimation

The slope of the lines drawn through the **H**‐domain are not easily clusterable in their current form as a collection of scattered data points. We transform the scattered data points in **H**‐domain into a clusterable form by projecting the magnitude subdomain onto a unit hypersphere. The **H**‐domain magnitude data are projected onto a half‐unit hypersphere by normalizing the TF magnetometer data via the following equation.

(5) $$B^{*}\left(t,k\right)=\frac{\left|B\left(t,k\right)\right|}{\left\|B\left(t,k\right)\right\|}$$

**Figure 2:**
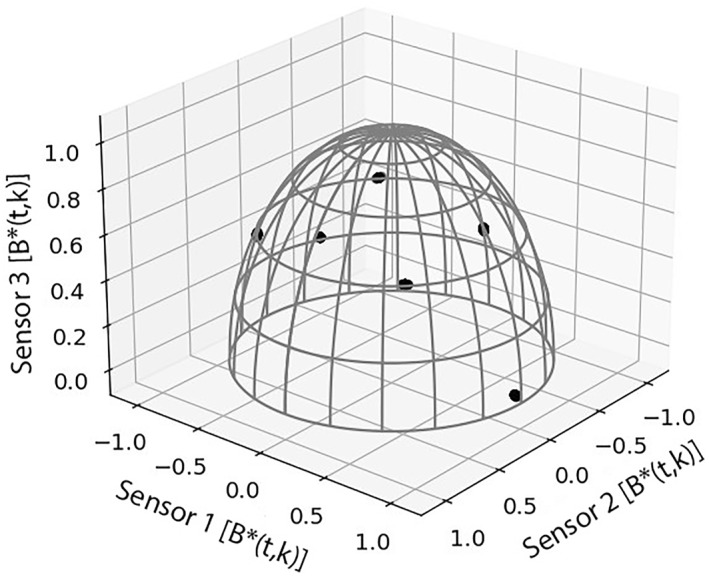
The scattered time‐frequency mixed signals in Figure 1 are projected onto a half‐unit hypersphere through normalization. The six scattered straight lines collapse into six compact clusters. The centroid of each cluster is proportional to each source signals' mixing vector in the mixing matrix, *K*, due to Equation 4.

When the scattered data points have been normalized, they collapse into compact clusters. This is illustrated by the projection of the scattered data points representing six computer generated signals in Figure 1 onto a half‐unit hypersphere in Figure 2. The centroid of a cluster is proportional to the mixing vector of a noise signal as defined in Equation 2.

The majority of the frequency space is filled with negligible energy points that will project randomly onto the unit hypersphere (Sun et al., 2016). We attempt to cleanse the data of these points using a magnitude filter. The filter is applied by finding the average signal magnitude and removing data points below a factor, *λ*, of the average signal magnitude. The magnitude filter is applied by removing data points that do not satisfy the following criterion:

(6) |B(t,k)|>λ⋅avg(|B(t,k)|)

The projected data points form tightly clustered groups on the unit hypersphere that allow us to discover the relative gain between noise signals at different magnetometers. However, we need to find the relative phases between noise signals of magnetometers at different positions. To account for this we join each projected TF point to its relative argument. The relative argument is defined by the following transformation:

(7) $$\arg\;B\left(t,k\right)=\left\{\;\arg\;B_{j}\left(t,k\right)-\arg\left(B_{0}\right.\left(t,k\right)|j\in \left[0,m\right]\;\right\}$$

Using the result of Equation 7, we define a new data format, *H*(*t*,*k*), by concatenating the projected magnitude data with the argument of the TF data.

(8) $$H\left(t,k\right)=\left(B^{*}\left(t,k\right),\;\arg\left(B\right.\left(t,k\right)\right)$$

**Figure 3:**
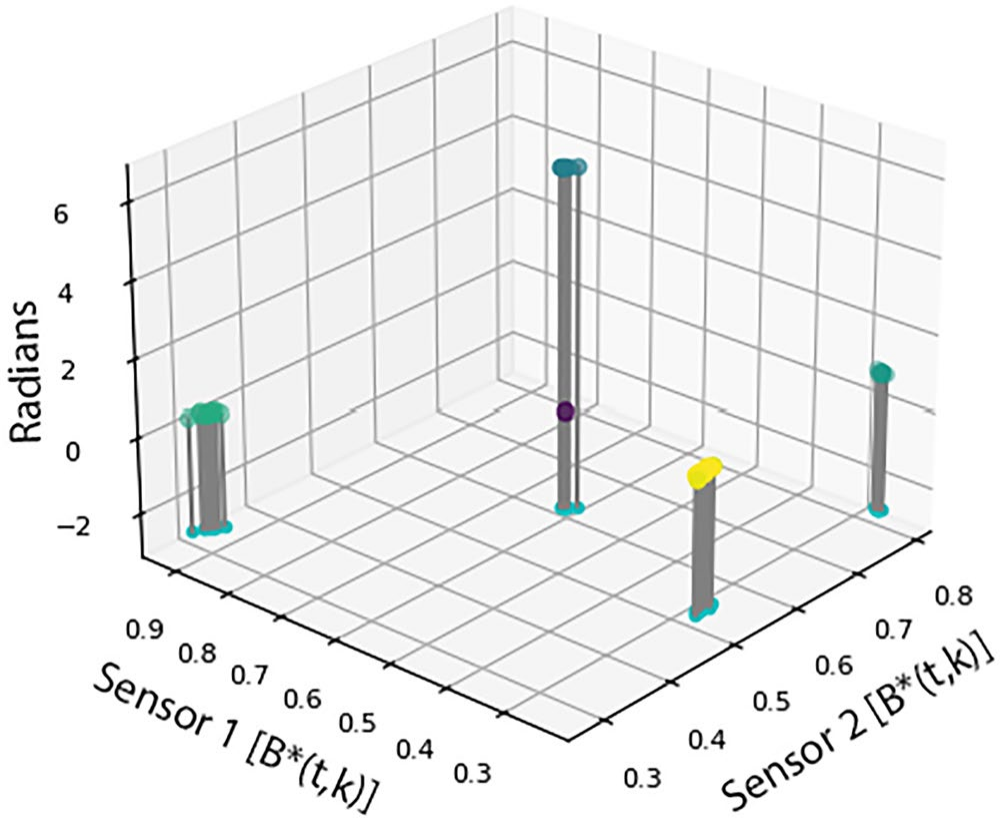
An illustration of noise signals in the full **H**‐domain for a two magnetometer system. The horizontal axes represent the magnitude of the time‐frequency magnetometer signals projected onto a unit hypersphere. The vertical axis represents the relative argument of Sensor 2 in radians as defined by Equation 7. The data points are projected onto a plane at *Z* = −2.5 to distinguish the difference in magnitudes. The phase and magnitude of each noise signal at each magnetometer is discovered by clustering the data in this format.

The magnetometer data, *H*(*t*,*k*), are now in a format that can be clustered to discover the gain and phase of each signal described in the mixing matrix, *K*. Figure 3 shows an example of two magnetometer signals transformed into the **H**‐Domain.

Now that the projected magnitude and relative phases are joined, a variety of clustering algorithms can be applied to find the mixing matrix, *K*. In this work, we use the Density Based Spatial Clustering for Applications with Noise (DBSCAN) algorithm because it does not require user input to discern the number of clusters present, and it will ignore noise points (Ester et al., 1996). As a result, the number of noise signals does not need to be defined prior to the application of DBSCAN. DBSCAN has two essential parameters, *eps* and *minPts*, that allow this functionality. The maximum distance for two points to become neighbors is the value, *eps*. If a point has *minPts* number of neighbors, it is called a core point. Core points are used to define each cluster. If a point is more than *eps* distance away from any point in a cluster, it is labeled as noise. We use DBSCAN to cluster *H*(*t*,*k*) and use each cluster's centroid as the noise signal's mixing vector. Once the mixing vector of each noise signal is known, the mixing vectors are joined to form the mixing matrix, *K*. The mixing matrix is used to separate the noise signals from the ambient magnetic field via compressive sensing.

### 2.3 Signal Reconstruction

Compressive sensing is a method used to reconstruct sparse signals with a sampling rate below two times a signal's bandwidth (Baraniuk, 2007). Reconstructing a signal of length *N* from a sampled signal of length *M*, where *M* < *N*, is an analogous problem to UBSS. Ordinarily, the system *b* = *Ks*, where *K* is a wide matrix, has infinitely many solutions because if *b* = *Ks* is a solution, *b* = *K*(*s* + *s* *′*) is also a solution for any vector *s*′ in the null space of *K*. Compressive sensing can exactly recover sparse signals and approximate near‐sparse signals through minimizing the L1 norm of S with respect to *b* − *Ks* < *ɛ*. The algorithm works with O(*N* ^3^) complexity.

We use CVXPY, A Python‐Embedded Modeling Language for Convex Optimization, to reconstruct the signals with the estimated mixing matrix, *K* (Diamond & Boyd, 2016). The formulation used to recover the signal, *s*, from *b* is:

(9) $$\begin{array}{cccc} & \text{Minimize} & & w^{T}|s|\\ & \text{Subject}\;\text{to} & & Ks=b \end{array}$$

Traditionally, compressive sensing minimizes the L1 norm of the source signals, ‖*s*‖_1_, with respect to Ks = *b* in order to recover the source signals. Instead of minimizing the L1 norm, we utilize a weighted L1 norm defined by the weighting vector, $w={\left[w_{1},\;1,\;1,\;\ldots ,\;1\right]}^{T}$, where *w*_1_ ≥ 1. The parameter, *w*_1_, is multiplied with the ambient magnetic field signal, *s*_1_, in order to deter the attribution of energy from other noise signals to it. In the case that the source signals, *s*, are not sparse at a TF bin, the additional weight increases the cost of attributing energy from other signals to the ambient magnetic field, *s*_1_. The optimal value of the weight, *w*_1_, depends on the signature of noise signals. Candès et al. (2008) apply a similar approach by iteratively adjusting the weight of each signal with respect to the magnitude of the signal. In this work, we found the optimal weight, *w*_1_, experimentally by comparing the reconstructed signal, ${\hat{s}}_{1}$, to the true signal, *s*_1_.

This system defined in Equation 9 is solved using the Embedded Conic Solver (ECOS) (Domahidi et al., 2013). The ECOS algorithm is a convex optimization algorithm that transforms the problem defined in Equation 9 into a Second Order Cone Problem (SOCP). SOCP problems are convex optimization problems that minimize linear functions with respect to second order cone constraints (Alizadeh & Goldfarb, 2003). The ECOS algorithm applies an interior point solver to converge on the sparse solution of the problem defined by Equation 9.

## 3 Experimental Data and Results

We test the proposed method of signal and noise separation through two experiments. The first experiment demonstrates the separation of SWARM magnetic field data from computer‐simulated signals using virtual magnetometers. The second experiment demonstrates the separation of SWARM magnetic field data from real magnetic noise signals generated with copper coils. The coil‐generated magnetic fields were measured using the PNI RM3100 magnetometer and a mock CubeSat described by Deshmukh et al. (2020).

**Figure 4:**
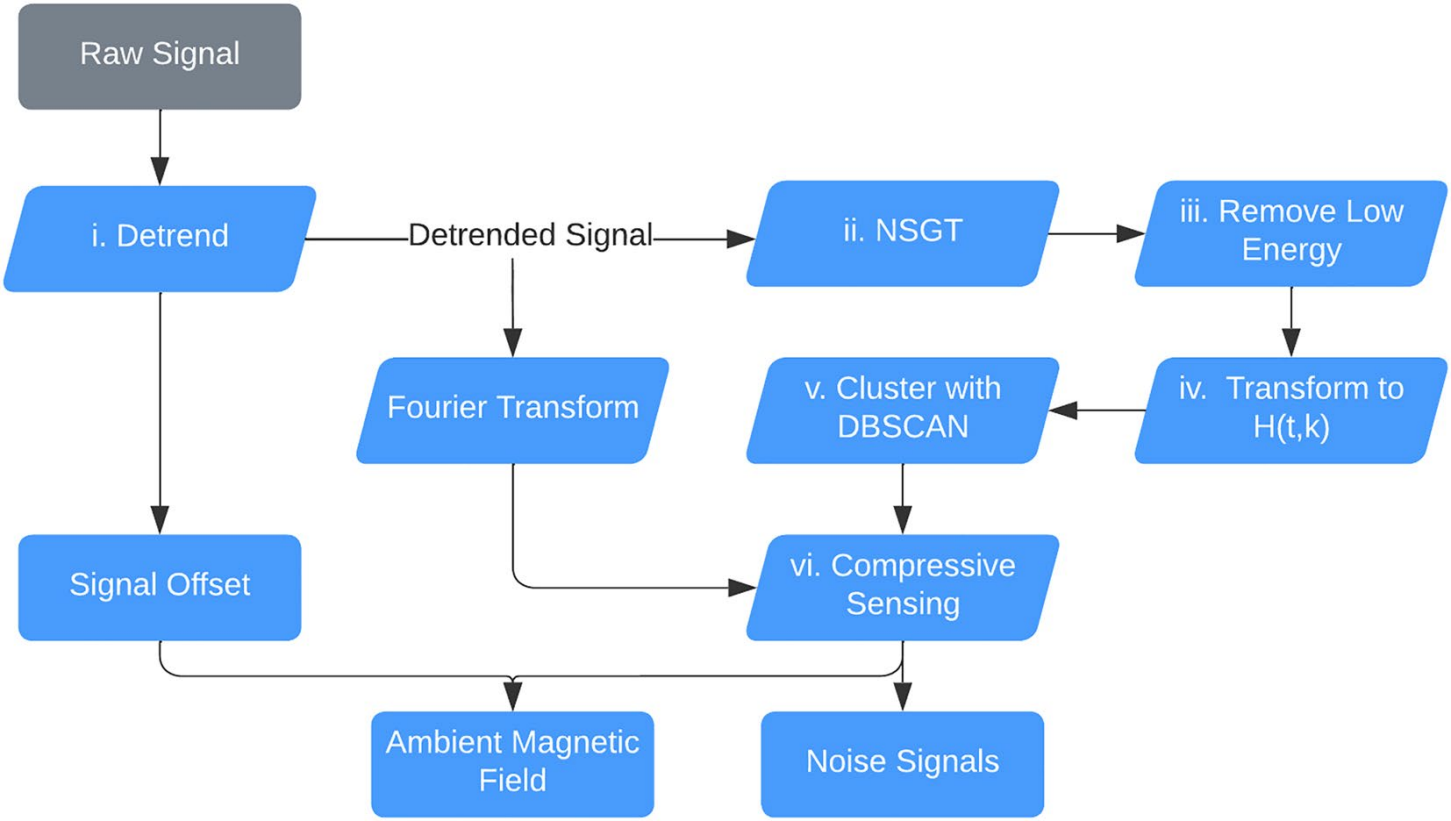
Flow of processes involved in using cluster analysis to discover noise signals and compressive sensing to separate the ambient magnetic field from noise signals.

Figure 4 details the process of identifying noise signals and reconstructing the ambient magnetic field. First (i), the signal offsets are subtracted to center the signals around 0 nT. Second (ii), the signals are transformed into the TF domain using the NSGT to increase signal sparsity. Third (iii), low energy points are filtered out using Equation 6. Fourth (iv), the signals are transformed into *H*(*t*,*k*) by projecting the magnitude, |*B*(*t*, *k*)| onto the unit hypersphere and concatenating it with the phase, arg  *B*(*t*, *k*), via Equations (5), (7) and 8. Fifth (e), the data, *H*(*t*,*k*), are clustered using DBSCAN and the cluster centroids are found. Finally, in the last step (vi), compressive sensing is used to reconstruct the ambient magnetic field. The minimum magnitude, *λ* in step (iii), and the parameters eps and MinPts in step (v) may need to be adjusted depending on the length and magnitude of the signals being analyzed.

We evaluate the separation of noise signals via three metrics. The metrics are calculated point‐wise using the reconstructed signal, *x* and the true signal, *y*, over *N* data points. The first metric is the Pearson Correlation Coefficient. This measurement gives the covariance between the normalized input and recovered signals.

(10) $$\rho =\frac{\sum_{i=0}^{N-1}\left(x_{i}-\bar{x}\right)\left(y_{i}-\bar{y}\right)}{\sqrt{\sum_{i=0}^{N-1}|\left(x_{i}-\bar{x}\right){|}^{2}\sum_{i=0}^{N-1}|\left(y_{i}-\bar{y}\right){|}^{2}}}$$

The second metric evaluated is the root mean squared error (RMSE). This metric is proportional to the magnitude of the squared error. As a result, the RMSE is very sensitive to large errors.

(11) $$RMSE=\sqrt{\frac{\sum_{i=0}^{N-1}{\left(x_{i}-y_{i}\right)}^{2}}{N}}$$

The final metric is the normalized RMSE (NRMSE). This metric yields the RMSE as a percentage of the magnitude of the signal being measured. It is used to compare the relative error between signals on different orders of magnitude. We calculate the NRMSE by dividing the RMSE of the signal by the max amplitude of the absolute value of the true, detrended signal, $|y-\bar{y}{|}_{\max}$.

(12) $$NRMSE=\frac{RMSE}{|y-\bar{y}{|}_{\max}}$$

### 3.1 Experiment 1: Computer Simulation

In this experiment, we use four simulated noise signals, *s*(*t*) ⊃ [*s*_2_(*t*), *s*_3_(*t*), *s*_4_(*t*), *s*_5_(*t*)], and three virtual magnetometers *b*(*t*) = *Ks*(*t*) = [*b*_1_(*t*), *b*_2_(*t*), *b*_3_(*t*)]. The signal, *s*_1_(*t*), is residual magnetic field data created by subtracting data generated by the IGRF model from SWARM magnetic field data. This process leaves only magnetic perturbations present in the magnetosphere. The magnetic perturbation data we use were measured by the SWARM A satellite on 17 March 2015 between 8:53 and 8:55 UTC. This part of the orbit passes between the 69th and 76th parallel south and was selected to capture perturbations in the southern auroral zone. The proposed algorithm detailed in Figure 4 is tested on 100 s of data, although it may be applied to a signal of any length provided that there are enough data points to cluster. The signals are combined through the complex mixing matrix in Equation 13 with phases given in radians.

(13) $$K=\left[\begin{array}{ccccc} 1∠0 & 0.99∠0 & 0.09∠0 & 0.70∠0 & 0.02∠0\\ 1∠0 & 0.09∠\pi & 0.99∠0 & 0.70∠0 & 0.05∠\pi \\ 1∠0 & 0.12∠\pi & 0.12∠\pi & 0.13∠\pi & 0.99∠\pi \end{array}\right]$$

**Figure 5:**
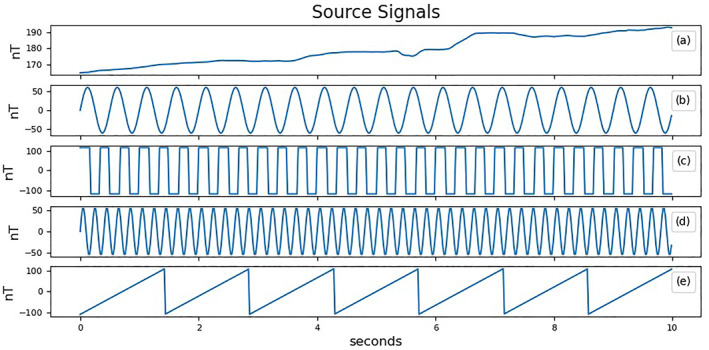
10 s of four source signals used to simulate spacecraft noise and one signal to simulate the ambient magnetic field. (a) The ambient magnetic field signal using SWARM A data starting from 17 March 2015 at 8:53 UTC. (b) A 2 Hz sine wave with amplitude of 50 nT. (c) A 3 Hz square wave with a magnitude of 100 nT. (d) A sine wave with a frequency of 5 Hz and amplitude of 50 nT. (e) A sawtooth wave with an amplitude of 110 nT and frequency of 0.7 Hz.

The values in the first column represent the ambient magnetic field signal which appears identically at every magnetometer. Figure 5 shows the five source signals used in this simulation. Two of the noise signals are sine waves with frequencies of 2 and 5 Hz. Sine waves are sparse signals that can be represented by a single point in the frequency domain. This makes them easily identifiable by cluster analysis. The two remaining noise signals used are a sawtooth wave with a frequency of 0.7 Hz, and a square wave with a frequency of 3.0 Hz. These signals inhabit a broad frequency spectrum and diminish the sparsity of the mixed signals.

**Figure 6:**
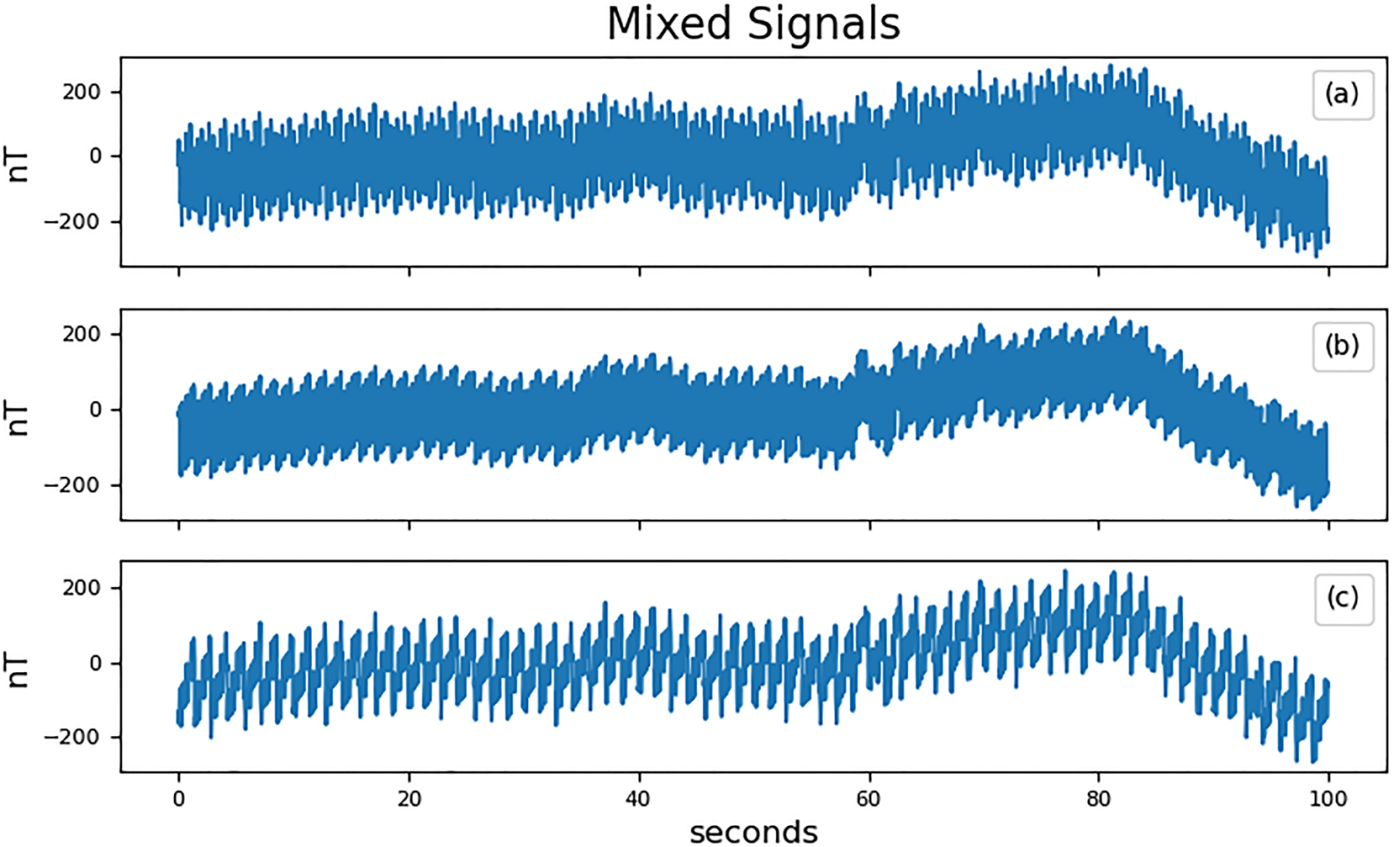
Plots (a, b, and c) show one hundred seconds of three magnetometer signals, *b*(*t*), created by mixing the five source signals in Figure 5 though the mixing matrix defined in Equation 13.

The signals are combined in the mixing system *b*(*t*) = *Ks*(*t*) with the mixing matrix *K* from Equation 13. The resulting signals are sampled by the virtual magnetometers at a rate of 50 samples per second. Different noise signals, such as noise generated by reaction wheels, may have higher frequency components and require a higher sampling rate in order to avoid aliasing (Miles et al., 2016; Pope et al., 2011). A random normal signal with a standard deviation of 6 nT is added to each virtual magnetometer in order to simulate instrument noise. This noise level corresponds to the rated instrument resolution of the PNI RM3100 magnetometer at 50 Hz used in Experiment 2. Figure 6 shows the sampled signals.

Following the procedure in Figure 4, the signals were detrended and transformed into the TF domain using the NSGT. The NSGT is a type of constant‐Q transform, so it requires the parameter *Q* which specifies window size. In this experiment, we used *Q* = 10 and a lower frequency bound of 30 mHz. In step (iv), low energy points were removed using a *λ* = 0.5. The resulting data were transformed into *H*(*t*,*k*) and clustered by DBSCAN with parameters *eps* = 0.3 and *MinPts* = 4. These parameters were optimized experimentally using trial and error, however it may be possible to automate parameter selection based on the signals being analyzed. With this configuration, DBSCAN discovered the five clusters corresponding to each noise source. The clusters, shown below in the columns of $\hat{K}$, closely match the original mixing matrix.

(14) $$\hat{K}=\left[\begin{array}{ccccc} 1∠0 & 0.99∠0.00 & 0.697∠0.00 & 0.10∠0.00 & 0.05∠0.00\\ 1∠0 & 0.10∠-0.02 & 0.697∠0.14 & 0.99∠0.06 & 0.14∠3.10\\ 1∠0 & 0.12∠-3.10 & 0.135∠3.14 & 0.12∠-3.10 & 0.98∠-3.16 \end{array}\right]$$

**Figure 7:**
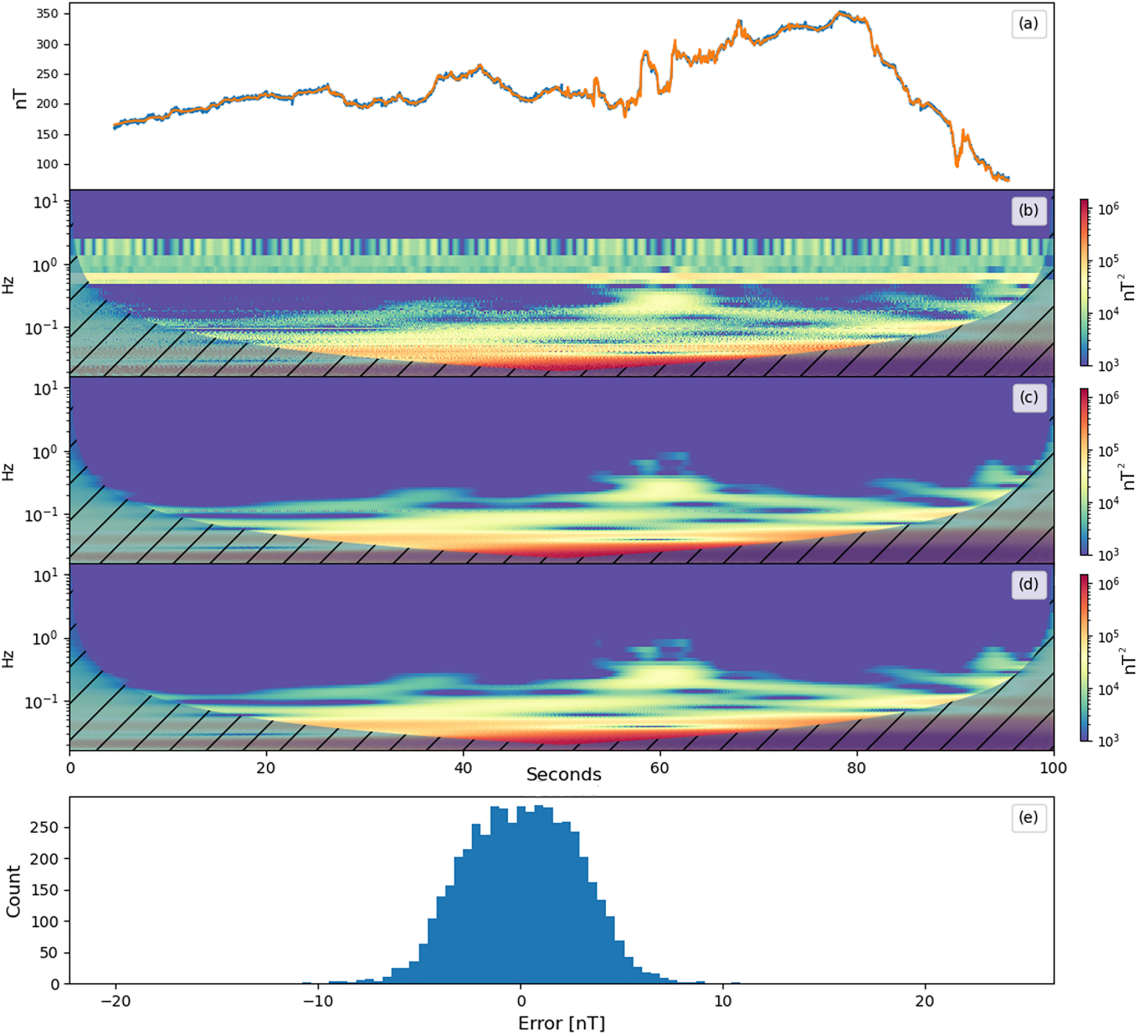
The top plot (a) shows the cleaned magnetometer signal in blue with the ambient magnetic field signal overlayed in orange. Plot (b) shows a spectrogram of the uncleaned signal from magnetometer (a) in Figure 6. Plot (c) shows a spectrogram of the reconstructed ambient magnetic field signal. Plot (d) shows a spectrogram of the true ambient magnetic field signal. The spectrograms were created using wavelet analysis. The shaded areas indicate where the wavelet does not produce valid results. The bottom plot (d) shows a histogram of the signal reconstruction error, $s_{1}-{\hat{s}}_{1}$.

Finally, in step (vi), the mixed signals were separated by compressive sensing using the recovered mixing matrix, $\hat{K}$, in Equation 14. The data, *H*(*t*,*k*), are discarded and the raw Fourier transform of the mixed signals are separated by applying the ECOS algorithm to the problem defined in Equation 9 with a weight of *w*_1_ = 1.5. The reconstructed SWARM perturbation signal is shown in Figure 7, as well as a histogram of the reconstruction error and spectrograms of the noisy, cleaned, true SWARM signal. A breakout of the reconstructed noise signals is included as Figure S1.

The reconstructed ambient magnetic field signal resembles the original signal with some additional error. In order to evaluate the reconstruction noise, the Pearson correlation coefficient, RMSE, and NRMSE of each source signal are calculated. The ambient magnetic field was reconstructed with a RMSE of 2.75 nT. The results for the reconstruction of each source signal are shown in Table 1. The experiment was repeated without the addition of the 6 nT instrument noise to evaluate the effect of the random noise on the total reconstruction error.

**Table 1:** Summary of Experiment 1 Results

	Metric	SWARM	Sine A	Square	Sine B	Sawtooth
With noise	*ρ*	0.9988	0.9934	0.9983	0.9941	0.9982
	RMSE	2.75 nT	4.11 nT	5.77 nT	6.39 nT	2.54 nT
	NRMSE	1.21%	8.23%	5.77%	6.39%	5.35%
Without noise	*ρ*	0.9988	0.9927	0.9987	0.9941	0.9974
	RMSE	2.84 nT	4.33 nT	7.06 nT	6.38 nT	3.42 nT
	NRMSE	0.81%	8.68%	7.06%	6.38%	7.21%

### 3.2 Experiment 2: Magnetic‐Coil‐Generated Signal Separation

In this experiment, we demonstrate the utility of the proposed algorithm on real magnetic field data. We use three PNI RM3100 magnetometers to record copper coil‐generated noise signals. Four copper coils are driven by signal generators to create the source signals, *s*(*t*) ⊃ [*s*_2_(*t*), *s*_3_(*t*), *s*_4_(*t*), *s*_5_(*t*)]. The signals are combined in the unknown mixing system, *b*(*t*) = *Ks*(*t*) = [*b*_1_(*t*), *b*_2_(*t*), *b*_3_(*t*)]. The SWARM residual magnetic field data, which is used in experiment one, is added to each magnetometer recording to generate the ambient magnetic field signal, *s*_1_(*t*).

**Figure 8:**
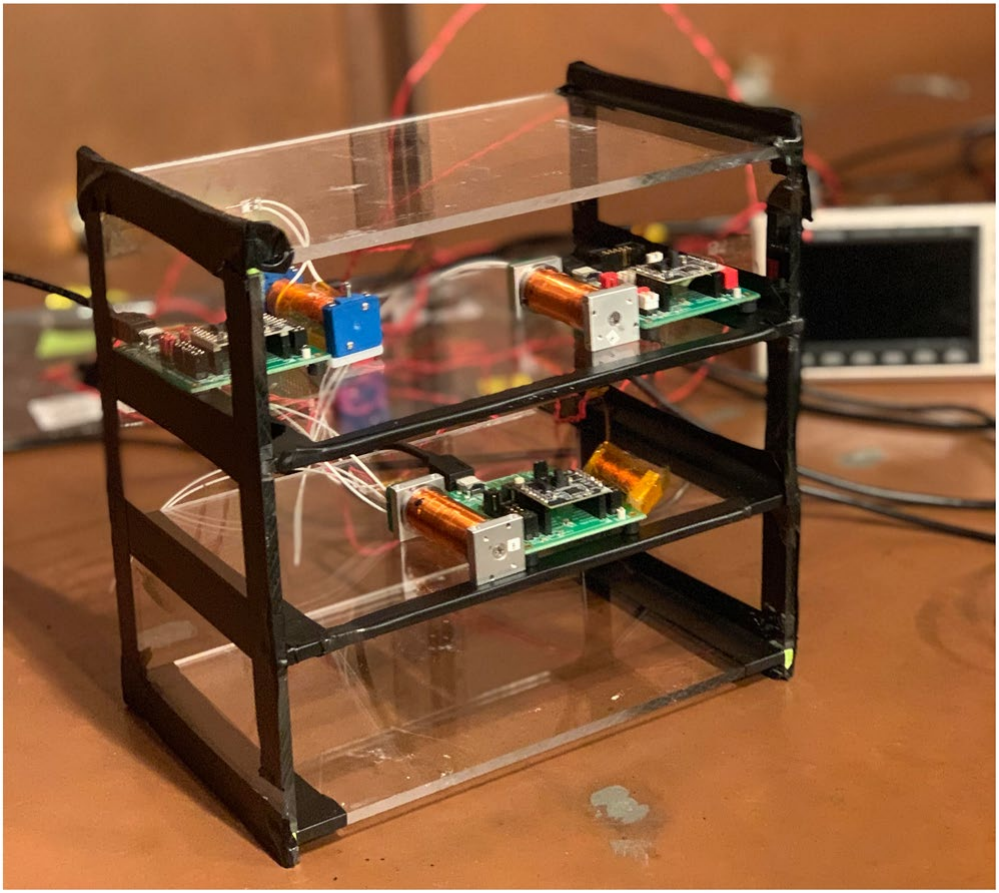
Mock CubeSat Apparatus with three PNI RM3100 magnetometers and four copper coils driven by signal generators. The magnetometers are placed within the mock CubeSat. In this study, we do not examine the effect of surface mounted sensors or sensors placed on a boom. The Apparatus is placed inside a mu‐metal lined copper room that acts as a large magnetic shield can.

The proposed algorithm detailed in Figure 4 is tested on 100 s of recorded data. The signals, *s*_2_(*t*) and *s*_3_(*t*), are sine waves with frequencies of 0.4 and 0.8 Hz. The signals, *s*_4_(*t*) and *s*_5_(*t*), are square waves with frequencies of 1 and 2 Hz. The three PNI RM3100 magnetometers and four copper coils are placed on the CubeSat apparatus as shown in Figure 8. Due to the location and orientation of the four copper coils and three magnetometers, each noise signal will appear at each magnetometer with a different magnitude and magnetic latitude induced phase. Additionally, this experiment was performed in a copper room lined with mu‐metal in order to screen out magnetic fields from the surrounding environment.

**Figure 9:**
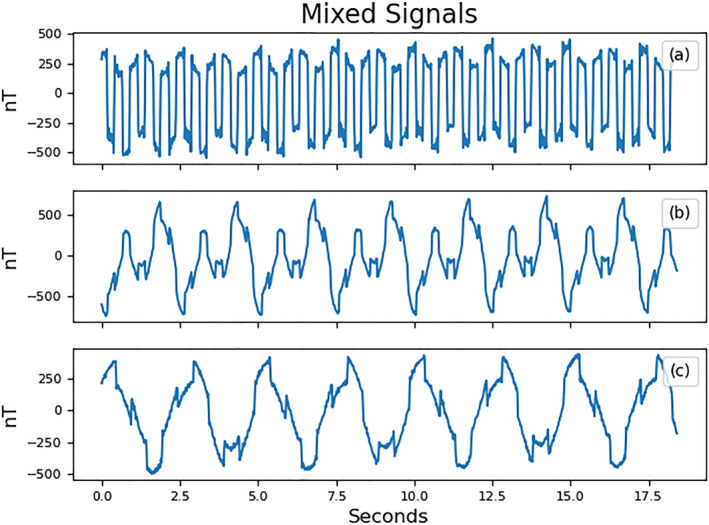
Plots (a, b, and c) show 18.5 s of three mixed signals recorded by PNI RM3100 magnetometers' *z*‐axis. The five signals present are two sine waves, two square waves, and the added residual magnetic field data. The noise signals have amplitudes between 50 and 500 nT compared to the ambient magnetic field signal with a max amplitude near 300 nT.

The PNI RM3100 is a magneto‐inductive magnetometer that measures the magnetic field by counting hysteresis loops with a comparator circuit, called a Schmitt Trigger, in an ASIC. The ASIC records magnetic field measurements by adding to a register every time the Schmitt trigger is saturated. This measurement renders the magnetic field when integrated with respect to time. The ASIC has a cycle count register that controls how many clock cycles pass between integrations. The error of the magnetometer will change with respect to the cycle count. In this experiment, each magnetometer is sampled at a rate of 50 Hz with a cycle count of 200 cycles. The PNI RM3100 is rated to have a resolution of 6 nT in this configuration. The mixed signals recorded by the PNI RM3100 magnetometers are shown in Figure 9 below.

The proposed algorithm was run on data from the magnetometers' *z*‐axis following the same steps as in Figure 4 and Section 3.1. The signals were detrended and transformed into the TF domain using the NSGT with a quality factor of *Q* = 20 and a lower frequency bound of 30 mHz. In step 4, low energy points were removed using a *λ* = 2.5. The resulting data were transformed into *H*(*t*,*k*) and clustered by DBSCAN with parameters *eps* = 0.4 and *MinPts* = 4. DBSCAN discovered the following five clusters shown below in the columns of $\hat{K}$.

(15) $$\hat{K}=\left[\begin{array}{ccccc} 1∠0 & 0.023∠0 & 0.22∠0 & 0.93∠0 & 0.02∠0\\ 1∠0 & 0.55∠1.31 & 0.97∠3.09 & 0.35∠3.04 & 0.04∠6.04\\ 1∠0 & 0.79∠4.58 & 0.001∠2.94 & 0.15∠0.255 & 0.82∠2.84 \end{array}\right]$$

**Figure 10:**
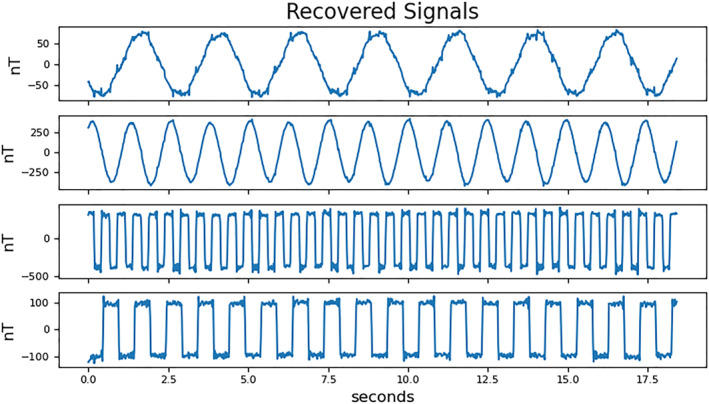
Reconstructed Sine and Square wave signals from 50 Hz mixed signals in Figure 9.

The PNI RM3100 magnetometer was experimentally found to have a lower noise floor when sampled at a higher rate and decimated to a lower rate versus only being sampled at a lower rate. We evaluated this effect by reconstructing the original 50 Hz data in step 6, then downsampling the reconstructed ambient magnetic field signal to 10 Hz, 1 Hz, and averaging the data with a moving mean (*N* = 10). The magnetometer signals were downsampled by applying an eighth order Chebyshev type I anti‐aliasing filter and resampling the resulting signal. The mixed signals were separated via weighted compressive sensing using a weight of *w*_1_ = 3. The four noise signals reconstructed from the 50 Hz raw data are shown in Figure 10.

**Figure 11:**
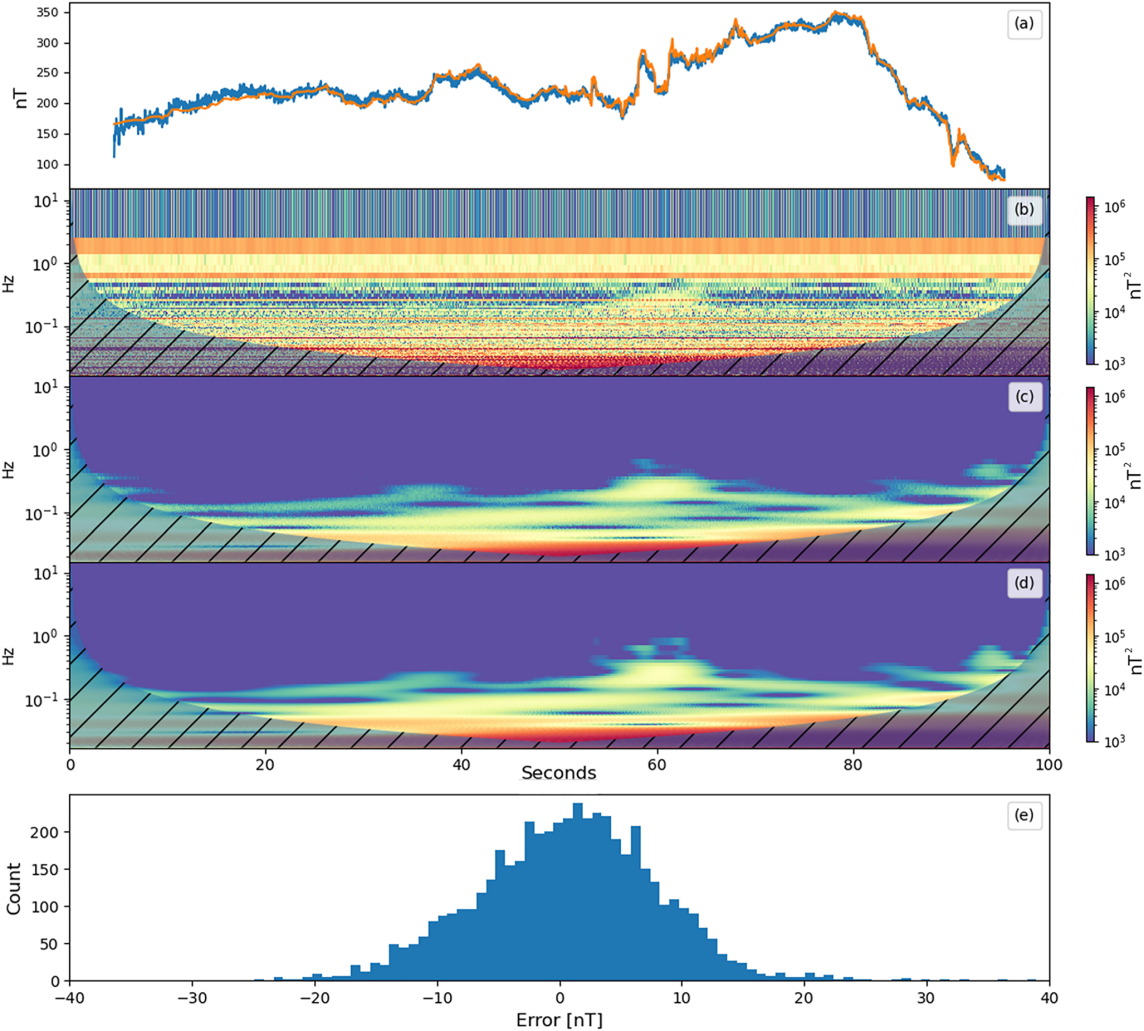
The top plot (a) shows the cleaned magnetometer signal in blue with the ambient magnetic field signal overlayed in orange. Plot (b) shows a spectrogram of the uncleaned signal from magnetometer (a) in Figure 9. Plot (c) shows a spectrogram of the reconstructed ambient magnetic field signal. Plot (d) shows a spectrogram of the true ambient magnetic field signal. The spectrograms were created using wavelet analysis. The shaded areas indicate where the wavelet does not produce valid results. The bottom plot (d) shows a histogram of the signal reconstruction error, $s_{1}-{\hat{s}}_{1}$.

The reconstructed coil‐generated signals closely resemble square and sine waves with some additional noise. The recovered residual magnetic field data are shown in the top plot of Figure 11. The recovered signal is overlayed with the true residual magnetic field signal. The residual data in Figure 11 were reconstructed using the mixed signals sampled at the full 50 Hz cadence. The plots below show the reconstructed signal, spectrograms of the noisy, cleaned, and true SWARM signal created using wavelet analysis, and a histogram of the signal reconstruction error.

The reconstructed signal closely follows the true geomagnetic perturbation signal with some high frequency noise present. As a result of the geomagnetic field signal being artificially inserted into the magnetometer readings, we are able to calculate the RMSE and Pearson Correlation Coefficient with respect to the original signal. The results for the original, decimated, and moving‐mean signals are shown in Table 2. These results are also compared to the uncleaned magnetometer data from magnetometer (a) in Figure 9.

**Table 2:** Summary of Experiment 2 Results

	Metric	50 Hz	10 Hz	1 Hz	Moving mean (*N* = 10)
Recovered signal	*ρ*	0.9947	0.9958	0.9952	0.9955
	RMSE	7.94 nT	7.23 nT	7.41 nT	7.45 nT
	NRMSE	2.26%	2.08%	2.13%	2.11%
Noisy signal	*ρ*	0.2126	0.2286	0.9139	0.2871
	RMSE	328.08 nT	300.53 nT	30.63 nT	239.33 nT
	NRMSE	93.31%	86.69%	8.84%	68.0%

## 4 Discussion

In this study, we introduced a signal processing algorithm based on UBSS and demonstrated the separation of magnetic noise from geomagnetic field data. In the first experiment, we separated four simulated noise signals from SWARM residual magnetic field data. The noise signals contained both sparse sine wave signals and wideband sawtooth and square wave signals. The algorithm was able to restore the residual magnetic field signal with a correlation coefficient of *ρ* = 0.9988 and RMSE of 2.75 nT. When the experiment was repeated without artificial instrument noise, the algorithm reconstructed the ambient magnetic field signal with a RMSE of 2.84 nT. In the second experiment, we created four magnetic noise signals using copper coils to generate real magnetic field data and placed PNI RM3100 magnetometers within the bus of a mock CubeSat apparatus. The same SWARM magnetic residual data were artificially inserted into the magnetometer measurements. This experiment mimicked the computer‐simulated experiment, with two sparse noise signals and two wideband noise signals. At a sampling rate of 50 Hz, the ambient magnetic field signal was reconstructed with a RMSE of 7.94 nT as opposed to 2.75 nT in simulation. The signal separation algorithm was executed using several additional preprocessing techniques such as decimating the sampling rate and applying a moving mean to the magnetometer data. A RMSE of 7.41 nT was achieved by decimating the sample rate to 1 Hz. At 1 Hz, the PNI RM3100 magnetometer is rated to have a measurement error of 2.7 nT due to instrument noise (Regoli et al., 2018). This result places the reconstruction error near the measurement resolution of the magnetometer. When the noisy magnetometer data were decimated, it reduced the RMSE of the signal measured by magnetometer (a) in Figure 9 from 328.1 to 30.6 nT. In contrast, the decimation of the ambient magnetic field signal reconstructed from the proposed algorithm did not significantly improve the RMSE. The reconstructed signal decimated to 1 Hz had an RMSE of 7.41 nT compared to 7.94 nT at 50 Hz, however, the UBSS algorithm was able to improve the RMSE by over 20 nT compared to simple downsampling. These results show that the proposed UBSS algorithm is effective at removing spacecraft noise from magnetic field data.

In general, it is not feasible to adaptively cancel spacecraft noise when a single magnetometer is used. Adaptive noise cancellation requires the removal of noise signals that are time variable. The use of a single magnetometer requires that spacecraft noise be carefully characterized before launch. Otherwise, a change in spacecraft behavior may require special maneuvers to re‐characterize noise signatures in situ (Miles et al., 2019). The use of multiple magnetometers allows for the discovery of noise signals through the comparison of magnetometer data. Sheinker and Moldwin (2016), Deshmukh et al. (2020), and Imajo et al. (2021) each propose algorithms for noise cancellation using multiple magnetometers. The algorithm proposed by Sheinker and Moldwin (2016) is effective at removing a single noise signal, but is not designed for multiple noise signals. Imajo et al. (2021) propose the use of ICA which is also limited by how many noise signals it can remove. BSS algorithms require that the number of source signals be less than or equal to the number of mixed signals. Spacecraft contain many electrical systems that could generate magnetic interference, so this condition is rarely met. For example, Pope et al. (2011) identified seven common types of noise signals on Venus Express, which is equipped with two magnetometers. The advantage of the proposed UBSS algorithm over Imajo et al. (2021) and Sheinker and Moldwin (2016) is that it can cancel noise signals in an underdetermined system. This means that there are more noise signals present than magnetometers. This property of the algorithm provides the flexibility necessary to be applied to many different spacecraft without prior characterization of spacecraft noise. The algorithm also does not require knowledge of magnetometer location and orientation, except that the axis of each magnetometer are aligned. Finally, Deshmukh et al. (2020) designed a state estimation algorithm to transform housekeeping data to magnetic noise signals. Housekeeping currents provide an incomplete mapping of the distribution of currents within a spacecraft. Additionally, housekeeping data are often sampled at a low cadence and may not have the appropriate bandwidth to identify higher frequency noise. The advantage of the proposed UBSS algorithm over this approach is that it is a blind signal processing algorithm. It requires no housekeeping data to identify and remove noise signals.

The proposed algorithm functions on the assumption that the noise signals are sparse, meaning that only one noise signal is present at a given frequency. Multiple noise signals may be active at the same time, however, if a signal is not sparse in the frequency domain, then its mixing vector cannot be accurately estimated by cluster analysis. Compressive sensing also requires sparsity in order to accurately reconstruct the separate signals. Compressive sensing can fully reconstruct sparse signals, and approximately reconstruct near‐sparse signals. In this work, we do not exhaustively explore the minimum sparsity required for accurate reconstruction of the ambient magnetic field.

The proposed algorithm requires that several parameters be set by the user. In this study, the parameters were manually selected based on the signals being analyzed, but this process could also be automated. The first parameter is the quality factor, *Q*. This parameter adjusts the window size used in the NSGT. We experimentally selected it, but it may be chosen based on the length of the signal being processed. The parameter, *λ*, is used to remove low energy noise signals. Data points that are below a fraction, *λ*, of the average energy data point are removed before clustering occurs. We selected this parameter by analyzing the data projected onto the half‐unit hypersphere in Figure 2, and visually observing if the signals were clusterable. If *λ* is too small, then the hypersphere will be completely filled with data points, and the noise signals will not be separable. If *λ* is too large, then small noise signals may not appear at all. Lastly, DBSCAN requires that two parameters, *eps*, and *MinPts*, be selected. The parameter, *eps*, represents the maximum distance allowed for two data points to be considered neighbors. The parameter, *MinPts*, represents the number of neighbors required for a data point to be considered a core. *MinPts* may be selected based on the length of signal being processed. A disadvantage of using NSGT and DBSCAN together is that more data points are created for higher frequency signals because the window size is altered based on frequency. Therefore, *MinPts* should be selected based on the lower frequency signals.

Most heliophysics missions require magnetic field accuracies of better than 1 nT (e.g., the NASA MMS mission (Russell et al., 2016)). Using the PNI RM3100 magnetometer, the algorithm reconstructed the ambient magnetic field signal with an RMSE of 7.94 nT. This error is near the expected measurement noise for the PNI RM3100 magnetometer at 50 Hz, indicating that the accuracy of the algorithm is limited to the total error budget of the magnetometer. Nevertheless, the experiments performed show the successful reconstruction of magnetic perturbation signals measured from within the bus of a mock CubeSat. These results demonstrate the utility of boomless CubeSats for scientific investigation of magnetic field phenomena in the geospace environment. In turn, the low cost of CubeSats enables the use of large constellations of small satellites to measure the geomagnetic field with high temporal and spatial resolution.

## 5 Conclusions and Future Work

In this study, we propose an algorithm for separating spacecraft generated magnetic noise from geomagnetic field data using multiple magnetometers. The algorithm does not require knowledge of the characteristics (location, orientation, amplitude, or spectral signature) and allows the number of noise sources to exceed the number of magnetometers (*n* > m). The algorithm identifies signals by looking at the relative gain and phase of the magnetometer data in the TF domain. If a noise signal is sparse in this domain, the relative gain and phase is found using cluster analysis. Following the same assumption of sparsity, the signal can be separated from the noisy data using the cluster centroids in compressive sensing.

The algorithm is designed for underdetermined systems in which there are more noise sources than magnetometers. An advantage of this approach is that the UBSS algorithm can be integrated onto any satellite since no prior characterization of noise signals is required. This design eases the assimilation of magnetometers into spacecraft designs by reducing the need for strict magnetic cleanliness requirements and long mechanical booms.

There are several avenues of future development for this algorithm. The most immediate step to be taken is for the selection of parameters to be automated. We present an algorithm to automate the noise cancellation process, but some rudimentary analysis is still required to select parameters for clustering and preprocessing. We think the selection of parameters could be entirely automated. Another avenue of development is to test the limits of the sparsity assumption. Sparsity is a very strict assumption that may not always be met. In this work, we tested the algorithm using several wideband signals. However, the threshold for minimum sparsity is unknown. This assumption can be examined through examining signals with partially overlapping spectra to find a point of failure. Finally, an interesting scenario to investigate is where several magnetometers are mounted within the bus of a spacecraft, but one magnetometer is mounted on a short boom, such as on the spacecraft Dellingr (Kepko et al., 2017). In this scenario, the measurements of one magnetometer may be more accurate than the others. It would be counterproductive if the reconstructed magnetometer signal had more noise than the signal measured by the magnetometer on the boom. It may be possible to account for this by designing a programmable ”trust” parameter at the compressive sensing stage. This parameter would indicate an elevated degree of trust in one magnetometer over the others.

In this work, we performed two experiments to validate the algorithm. The first experiment separated SWARM magnetic perturbation data from four computer‐simulated signals. The algorithm was able to reconstruct the ambient magnetic field signal with an RMSE near 3 nT and a correlation of *ρ* ≈ 0.9988. The reconstruction errors are less than the 6 nT intrinsic instrument noise that was added to each virtual magnetometer. The second experiment used real magnetic noise signals generated by copper coils, and the same SWARM geomagnetic field data. This experiment was able to separate four noise signals and reconstruct the background magnetic perturbation signal with a RMSE of 7.23 nT and a correlation of *ρ* = 0.9958 at a 10 Hz cadence.

These results show the potential of signal processing algorithms to identify and remove magnetic noise from spaceborne magnetometer data. The proposed algorithm diminishes the need to place a magnetometer on a boom or enables significantly shorter booms. This enables the possibility of low cost, boomless spacecraft to capture high fidelity magnetic field measurements.

## Supporting information

Figure S1

## Data Availability

The SWARM magnetometer data are available from https://swarm-diss.eo.esa.int under MAGx_HR in the Level 1B data products folder. The noise signals generated in simulation and in the laboratory are available on the University of Michigan Deep Blue data repository (https://doi.org/10.7302/bz6v-6q52).
