# Supplementary material for: Separation of Spacecraft Noise From Geomagnetic Field Observations Through Density‐Based Cluster Analysis and Compressive Sensing
Source: J Geophys Res Space Phys. 2022 Sep 15;127(9):e2022JA030757. doi: 10.1029/2022JA030757 (PMC9541872; doi:10.1029/2022JA030757)
Supplement: Supplementary file 1 — Figure S1 [file JGRA-127-e2022JA030757-s001.pdf]

Supporting Information for "Separation of Spacecraft Noise from Geomagnetic Field Observations through Density-Based Cluster Analysis and Compressive Sensing"

Alex Paul Hoffmann[1], Mark B. Moldwin[1]

[1]Climate and Space Sciences and Engineering, University of Michigan, Ann Arbor, Michigan

Contents of this file

1. Figure S1 to Section 3.1

Introduction

Figure S1.

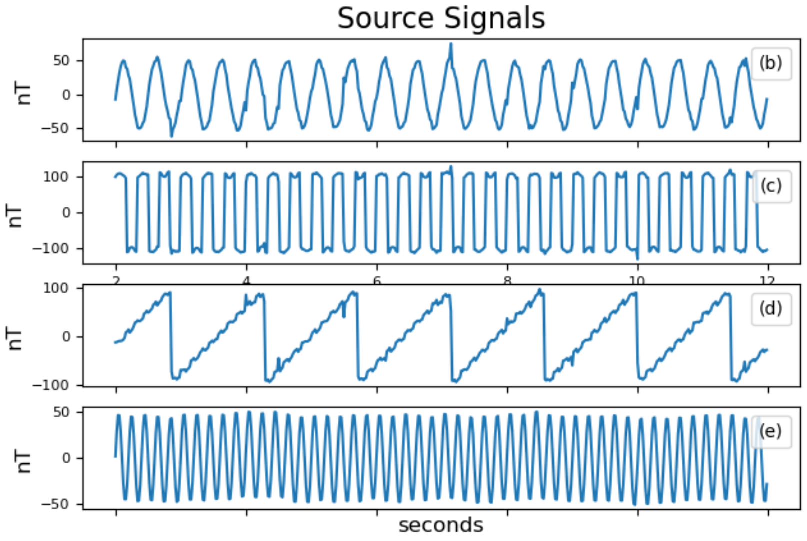

Figure 1. Plots (a), (b), (c), and (d) show the four reconstructed noise signals in experiment 1.
